# Prospective research on musculoskeletal disorders in office workers (PROMO): study protocol

**DOI:** 10.1186/1471-2474-7-55

**Published:** 2006-07-05

**Authors:** Stefan IJmker, Birgitte M Blatter, Allard J van der Beek, Willem van Mechelen, Paulien M Bongers

**Affiliations:** 1 Body@Work TNO VUmc, Research Center Physical Activity, Work and Health, VU University Medical Center, Van der Boechorststraat 7, 1081 BT Amsterdam, The Netherlands; 2 Department of Public and Occupational Health, Institute for Research in Extramural Medicine, VU University Medical Center, The Netherlands; 3 TNO Quality of Life, The Netherlands

## Abstract

**Background:**

This article describes the background and study design of the PROMO study (Prospective Research on Musculoskeletal disorders in Office workers). Few longitudinal studies have been performed to investigate the risk factors responsible for the incidence of hand, arm, shoulder and neck symptoms among office workers, given the observation that a large group of office workers might be at risk worldwide. Therefore, the PROMO study was designed. The main aim is to quantify the contribution of exposure to occupational computer use to the incidence of hand, arm, shoulder and neck symptoms. The results of this study might lead to more effective and/or cost-efficient preventive interventions among office workers.

**Methods/Design:**

A prospective cohort study is conducted, with a follow-up of 24 months. In total, 1821 participants filled out the first questionnaire (response rate of 74%). Data on exposure and outcome is collected using web-based self-reports. Outcome assessment takes place every three months during the follow-up period. Data on computer use are collected at baseline and continuously during follow-up using a software program.

**Discussion:**

The advantages of the PROMO study include the long follow-up period, the repeated measurement of both exposure and outcome, and the objective measurement of the duration of computer use. In the PROMO study, hypotheses stemming from lab-based and field-based research will be investigated.

## Background

Occupational computer use has become very common in the last decades. In 2003, the United States entailed over 77 million persons who used a computer at work [1]. In the European Union, over 88 million persons used a computer at work in 2002 [2]. Moreover, over 50 million European workers reported to use the computer at least half of their work time [3].

Recent large-scale surveys show one-year prevalences of hand, arm, shoulder and neck symptoms ranging from 24 to 44% among office workers [4-6]. The one-year incidence has been estimated to be 5 to 34%, depending on case definition and study population [4-8]. It should be noted that in most studies, both the prevalence and incidence of symptoms are higher in the neck-shoulder region than in the hand-arm region.

Given the large source population and the possible high incidence, a large number of office workers may be at risk for developing hand, arm, shoulder or neck symptoms. In addition, the costs related to hand, arm, shoulder, and neck symptoms (i.e. due to reduced productivity, sick leave, work disability, and medical consumption) are considerable. Blatter et al. [10] estimated the total costs at 2.1 billion euros per year for the Netherlands. Therefore, office workers, employers, and governments might benefit from improvements in the primary prevention of hand, arm, shoulder and neck symptoms.

The available epidemiological evidence suggests that hand, arm, shoulder and neck symptoms are associated with the duration of computer use and, in fact, increase steadily with each hour of computer use per day [11]. In addition, recent longitudinal studies suggest a dose-response relationship between the duration of mouse use and the incidence of hand-arm symptoms [4,6,7,12]. It should be noted that previous studies relied on self-reports for the measurement of the duration of computer use. However, the use of self-reports may lead to overestimation of the duration of computer use, which might result in misclassification [13-16]. Misclassification might bias the risk estimate and hamper the correct classification of office workers at risk for prevention purposes.

Despite the available evidence, controversy exists in the scientific and public media on the explanation of the current prevalence and incidence of hand, arm, shoulder and neck symptoms among office workers. The contribution of occupational mechanical exposure (i.e. duration of computer use, working postures, and computer design) to the incidence of hand, arm, shoulder and neck symptoms has received ample attention.

Advocates of the work-relatedness of hand, arm, shoulder and neck symptoms propose that occupational mechanical exposures contribute to a large extent to the incidence of musculoskeletal disorders. The symptoms are explained by local muscle, tendon or nerve injury, caused by overload of the musculoskeletal system [17-19]. In contrast, critics have contradicted consistent signs of muscle, tendon and nerve injury among patients reporting hand, arm, shoulder and neck symptoms [20]. In addition, the contribution of occupational mechanical exposure to the incidence of hand, arm, shoulder and neck symptoms has been criticized [21-23]. Alternative explanations for the incidence of hand, arm, shoulder and neck symptoms include, among others, poor lifestyle habits, poor psychosocial work context and sociological factors, including increased public awareness and a broad definition of work incapacity by the compensation system.

The main reason for designing the PROMO study (Prospective Research On Musculoskeletal disorders among Office workers) is that few longitudinal studies have been performed among office workers, and that no longitudinal study on risk factors has measured computer use objectively. The main study objective is to quantify the contribution of exposure to occupational computer use to the incidence of hand, arm, shoulder and neck symptoms among office workers. In the PROMO study, the term occupational computer use includes reading from the computer screen and the use of input devices: mouse use (i.e. clicking and moving the mouse) and keyboard use.

Exposure to occupational computer use can be defined in different ways. Most studies have operationalized exposure to computer use as the average (or cumulative) duration of computer use (or its constituents: mouse and keyboard use) over a certain time period. Other operationalizations include the cumulative number of keystrokes or mouse clicks, variation in computers use between days or weeks, and distribution of usage periods (i.e. number of breaks taken within a certain time period). In this study, exposure to computer use will be measured objectively with a software program, which is installed on the individual workstation. In addition, self-reports will be collected.

The second study objective is to quantify the relative contribution of various occupational and non-occupational risk factors. Information on the population attributable fraction of risk factors and on the identification of subgroups with high risk will contribute to the discussion on the potential of preventive interventions among office workers and possibly to the design of preventive interventions among office workers.

In summary, the PROMO study addresses the following research questions:

A. What is the relation between the exposure to occupational computer use and the incidence of hand-arm and neck-shoulder symptoms?

B. What is the relative contribution of occupational mechanical exposure, occupational psychosocial exposure, leisure time exposure and individual factors to the incidence of hand-arm and neck-shoulder symptoms among office workers?

### Hypotheses

With respect to research question A, we expect that hand-arm symptoms are more strongly related to the duration of computer use than neck-shoulder symptoms. Previous studies showed the strongest and most consistent associations for computer use with the incidence of hand-arm symptoms [4,6,7,13]. In addition, based on the same studies, we expect to find indications for a dose-response relationship between the duration of mouse use and the incidence of hand-arm symptoms.

By answering research question B, we will investigate the contribution of occupational computer use to the incidence of hand, arm, shoulder and neck symptoms, compared to the contribution of various other occupational and non-occupational risk factors. Firstly, we expect occupational computer use to be the strongest risk factor. Previous longitudinal studies, which included individual factors as well as estimates of occupational mechanical and psychosocial exposure, and leisure time exposure, have found the most consistent and strongest associations between the duration of mouse use and the incidence of hand-arm symptoms [4,6,7,13]. In addition, we expect computer use to be more strongly associated with hand-arm and neck-shoulder symptoms than ergonomic factors (i.e. working posture and workstation characteristics) [24]. If ergonomic factors have a causal contribution, one would expect that the association with hand-arm and or neck-shoulder symptoms would become stronger when exposed to longer durations of computer use. Besides occupational mouse use, we expect occupational psychosocial exposure to be an independent risk factor for neck-shoulder symptoms [25].

Secondly, we expect that low levels of leisure time physical activity contribute modestly, at most, to the incidence of hand-arm and neck-shoulder symptoms. Previous longitudinal studies among office workers failed to show an association between low levels of leisure time physical activity and hand-arm and neck-shoulder symptoms [6,7,9,13]. Workers exposed to high mental stress during work time and to low physical activity during leisure time were found to have an increased risk in one study [8]. However, confidence intervals were wide in this study. It should be noted that most studies among office workers have included only crude measures of leisure time exposure. In a longitudinal study among manual workers and office workers, a protective effect of sports activities on the incidence of hand, arm, shoulder and neck symptoms was reported [9]. However, specific leisure time activities might increase the risk of symptoms. Miranda and co-workers [27] reported an increased risk of incident shoulder symptoms when playing volleyball frequently. Thirdly, both female gender and previous symptoms have been reported frequently as risk factors among office workers in the published literature [female gender: [6-9,13,28]; previous symptoms: [4,5,28]]. We will explore whether these individual factors act as effect modifiers in the associations between occupational and/or leisure time exposure, and hand-arm and neck-shoulder symptoms. In addition, we aim to explore the role of the personality trait overcommitment in the incidence of hand-arm and neck-shoulder symptoms. A longitudinal and a cross-sectional study showed indications of an increased risk of hand-arm and neck-shoulder symptoms among overcommited workers [29,30]

## Methods/Design

### Study design

A prospective cohort study is conducted, with a follow-up of 24 months. Assessment of the health outcome (symptoms and disability due to symptoms) takes place at baseline and every 3 months during follow-up. Exposure data on computer use are collected continuously during the study period, while additional exposure data and information on individual characteristics are gathered at baseline and after one year of follow-up. Participation is voluntarily and participants signed informed consent. The study design was approved by the Medical Ethics Committee of the VU University Medical Center (VUmc).

### Recruitment of the study population

Companies with a source population of at least 500 office workers were invited to participate in the study. The study population was recruited from five different employers in the Netherlands: a brewery, a financial consultancy firm, a university, a transportation company, and an insurance company. A department within a company was included if the department had a computer network from which the software for recording the duration of computer use could be installed on individual workstations, and if at least three quarters of the employees fulfilled the inclusion criteria (see table 1).

Employees within included departments were informed about the study via distributed flyers. In addition, all these employees received an e-mail with information on study objectives and required effort for participation. As incentive for participation, workers were offered the choice between two options. Firstly, the donation of a small sum of money (20 eurocents) to a charity organization (i.e. Medecins Sans Frontieres) for each questionnaire they would fill out. Secondly, joining a lottery for a weekend holiday to a European capital. The latter option was only possible if they would fill out all the questionnaires during follow-up. Finally, a team of researchers visited the worksites and asked individual employees to participate in the study. At the same time, memo blocks were handed out as incentive. In total, 2461 out of 9161 (27%) approached employees signed informed consent. Out of these 2461 participants, 1821 (74%) filled out the first questionnaire.

### Data collection procedure

Data on exposure and outcome is collected using web-based self-reports. Participants receive an e-mail containing a link to a questionnaire. By request, they can fill out a hard copy of the questionnaire. In case of non-response, participants receive a maximum of two reminders by e-mail. In addition, data on computer use is collected objectively with a software program. Participants who leave their job during follow-up, receive a paper questionnaire to their home address in order to check symptom status and the possible contribution of symptom status to turnover. This information will be used to check if a healthy worker (selection) effect has occurred: symptomatic workers might leave the study more frequently than healthy workers, leading to biased associations.

### Assessment of exposure to computer use

Data on computer use are collected at baseline and continuously during follow-up using the software program WorkPace version 3.0 (Niche Software Ltd/ErgoDirect). The program has been installed from the central network on the individual computer of the participants. The program records computer use from the moment the participant has logged in to the network. Data storage takes place on the individual computer. Periodically (i.e. after being logged in to the network for 6 hours, or during log-off) the individual data file is sent to a dedicated and secured folder on the central network. Recording is continued if a person logs in to the network on another computer. Registration of keystrokes, mouse clicks and mouse movements are stored as cumulative totals per day. Thus, separate estimates for the duration of total computer use (including reading from the screen, mouse and keyboard use), mouse use and keyboard can be retrieved. Based on the duration of the time interval between two consecutive active events (i.e. keying, mouse clicking or mouse movements), the duration of keyboard, mouse, and total computer use, as well as the duration of breaks are calculated. If a participant hits a key, moves or clicks a mouse within 30 seconds of previously hitting a key, moving or clicking the mouse, this "inter-events period" (in seconds) is stored as a usage period of total computer use. If the threshold time of 30 seconds is exceeded, the elapsed time period between two usage periods is stored as a break from total computer use. The threshold time for mouse use is 5 seconds and for keyboard use it is 2.5 seconds. Cumulative totals for several usage and break periods are stored for every day separately.

Previous research has shown good agreement between the WorkPace estimate and systematic observation for the duration of total computer use. On group level, the average duration of total computer use estimated with WorkPace is within 10% of the average duration of total computer use estimated with systematic observation [15,31]

### Assessment of other occupational exposures

Self-reported data on duration of computer and mouse use, historical computer use, precision demands, use of break and exercise software, workstation characteristics and working postures while using keyboard and mouse are gathered at baseline and after one year of follow-up. Clarifying illustrations have been added to the questions on working postures for optimal validity. This questionnaire also contains questions on job tenure, job contract characteristics (e.g. working hours and working days), overtime work and work continuation during formal (lunch) breaks.

Occupational psychosocial stressors and perceived stress are measured using a translated version of the Effort-Reward Imbalance questionnaire [32] and the need for recovery scale from the Questionnaire on Perception and Judgment of Work [33,34]. In addition, the subscale decision authority from the Job Content Questionnaire is used [35]. Mental load is assessed by a subscale of the Questionnaire on Perception and Judgment of Work [33]. Job satisfaction is measured using a single item of the Questionnaire Work and Health [36]. In addition to baseline and one-year follow-up measurements, information on job satisfaction and rapid increases of general workload is collected every 3 months.

### Assessment of leisure time exposures

At baseline and after one year of follow-up, physical activity during leisure time is assessed by two questions. The first question focuses on the average number of days per week in the last 3 months with moderate intensity physical activity (i.e. causing increased breathing frequency) lasting at least 30 minutes in total per day. This is done to check whether physical activity public health recommendations are met [37,38]. The second question focuses on the average number of days per week in the last 3 months with high intensity physical activity (i.e. causing sweating), and lasting at least 20 minutes in total per day [40,41]. Activities during leisure time involving forceful or repetitive arm or hand movements (e.g. participation in strength training of the upper extremities and racket sports, and playing music instruments) and duration of total computer use during leisure time are assessed separately with a self-administered questionnaire.

### Assessment of individual characteristics

At baseline and after one year of follow-up, the personality trait overcommitment is measured within the Effort-Reward Imbalance questionnaire [32]. General health, age, gender, education, hand dominance (i.e. preferred hand for handwriting), body height and body weight are assessed with a self-administered questionnaire.

### Assessment of health outcomes

Every three months, data concerning symptoms (pain or discomfort) in the lower back, neck, shoulder, arm or hand during the past 3 months are gathered by means of a validated, modified version [41] of the Nordic Questionnaire [42]. Localization (anatomical region and side [left and or right]), duration and frequency of episodes of symptoms are recorded. The intensity of symptoms is measured using Von Korff scales [43]. Limitations in work or leisure time activities due to symptoms are measured using a Dutch translation of the scales that were used in the EPI-mouse study [44]. Data on the work-relatedness of symptoms and the presence of systematic disease or other causes of symptoms (e.g. traffic accident, burns, and rheumatic disease) are also gathered. Sick leave due to symptoms is measured by two questions that have shown adequate agreement with company records in back pain research: 97% specificity and 88% sensitivity [45]. In addition, long-term sick leave (i.e. longer than 6 weeks) is registered by the participating organizations or their occupational health service. Data on duration (days), frequency, level (full or partial sick leave) and diagnostic code are gathered. Participants who consult the occupational physician (OP) because of prolonged sick leave (i.e. 3 to 6 weeks) are diagnosed according to the Dutch guideline for the management of arm, neck and shoulder symptoms by occupational physicians [46]. All OPs connected to the participating organizations received training on diagnosing and coding hand, arm, shoulder and neck symptoms to reduce inter-observer variation in diagnosis and coding.

### Statistical analysis

#### Case definition

Subjects who report one or more symptoms in the hand-arm region and/or the neck-shoulder region during baseline or follow-up will only be labelled a case if they restrict their activities at home or at work during leisure time and/or used self-medication and/or visited a medical professional because of their symptoms. This definition is in concordance with the definition of the Health Council of the Netherlands, which states that hand, arm, shoulder and neck symptoms lead to limitations in daily functioning or to participation problems [47].

#### Episodes

Workers with and without symptoms at baseline will be followed during follow-up. In this study, data analysis is guided by the notion that hand, arm, shoulder and neck symptoms might be episodic and are recurrent in nature: symptoms are present at a certain time point, symptoms are absent for a certain time period afterwards and then may come back again. The implication for data analysis is that one subject may have more than one episode of hand, arm, shoulder or neck symptoms during the two years of follow-up. A separate episode in this study is defined by the presence of symptoms during a recall period of 3 months followed and preceded by a recall period of 3 months without symptoms. The transition from a symptom free period to an episode of symptoms will be modelled as the outcome variable. Time lags will be defined in order to ensure that exposure precedes the health outcome.

#### Statistical models

Relative risks and confidence intervals will be estimated using Poisson regression with robust error variance [48]. We will use both the general linear model (i.e. "basic regression") and its extension: Generalized Estimation Equations (GEE) (i.e. "basic regression for repeated measures"). In addition, we aim to contrast the findings of the above-mentioned analyses with models that can take into account random factors, and measurement errors: generalized linear latent and mixed models [49]. These models have great flexibility by combining the advantages of hierarchical regression models (i.e. analysing clustered data [e.g. repeated measures of the same subject]) and structural equation models (i.e. taking into account measurement errors). Finally, adjusted population attributable risks will be calculated, using adjusted relative risks and adjusted estimates of risk factor prevalence [50].

#### Statistical power

In a symptom free heterogeneous population of workers, the one-year incidence of neck-shoulder and hand-arm symptoms is expected to be 7.5% and 12.5%, respectively [4,6,7,9,13]. Consequently, the two-year incidence is expected to be 15% for hand-arm, and 25% for neck-shoulder symptoms. Further calculation will be made for hand-arm symptoms, because of the lower incidence of these symptoms in the working population. It is well known that hand-arm symptoms are prevalent in the general population, including people not or little exposed to computer use. Therefore, a two-year incidence for hand-arm symptoms of 10% among low exposed subjects and 20% among high exposed subjects seems reasonable. This difference in incidence between low exposed and high exposed subjects can be detected in logistic regression with a sample of 429 subjects [51]. To calculate sample size, we made the following assumptions: incidence at the mean for all factors in the model = 0.15, odds ratio determinant = 2.0, odds ratio confounder = 1.6, agreement between measured exposure and true score (validity) = 0.70, correlation between confounder and exposure (multi-collinearity) = 0.30, statistical power = 0.80 and alpha = 0.05. In addition, to be able to study potential effect modification by individual factors we need to at least double the sample size (860 subjects). We assume that about 20% loss to follow-up may occur per year (40% for 2 years), resulting in a recruitment of 1433 subjects at baseline.

In addition to the sample size calculation above, repeated outcome assessment might decrease the required sample size, since we expect that neck-shoulder and hand-arm symptoms are episodic in nature [52]. The required sample size is proportional to the number of outcome measurements and the intra class correlation (ICC) between (binary) outcome measurements [53]. Based on our one-year follow-up data we calculated ICC for nine repeated measurements [see for formula: [54], p.224]. We used the procedure xtlogit in the Stata software, version 7, to estimate between subject variance on five repeated measures. As a result, the required sample size for hand-arm symptoms decreased from 429 subjects to 204 subjects. From this, it follows that the actual sample size of the PROMO study should be sufficient for adequate statistical power, taking potential effect modification into account.

## Discussion

### Methodological considerations

The advantages of the PROMO study in comparison with studies published so far include the long follow-up duration (two years), the repeated measurements of both exposure and outcome, and the objective measurement of the duration of computer use. These features of our study will enhance the accuracy of risk estimates. In addition, the frequent exposure and outcome assessment will provide more insight in the time window(s) of relevant exposure effects. This issue has been identified as an important topic, but has up to now not received appropriate attention [55,56].

At the same time, observational studies, including the PROMO study, are threatened by several sources of bias. Selection bias might be present, since only one out of four workers, who were invited for the study, participated. Selection bias might hamper generalizibility of study findings to target populations. The generalizability of our findings is dependent on the distributions of effect modifiers in the target population, compared to our study population. An adjusted risk estimate in the absence of effect modification is highly generalizable, since it is by definition independent of the distribution of confounders in a target population. In the literature, few effect modifiers have been identified among office workers, making selection bias unlikely, at this moment.

Internal validity is threatened, as in most observational cohort studies, by (residual) confounding. In order to ensure that participants could fill out the baseline questionnaire within 30 minutes, we decided to restrict the item pool to the most relevant variables based on the available evidence [11,24,57,58]. We cannot be certain that we did not miss relevant variables. In addition, except for the duration of computer use, all other exposures are measured by self-report. It is known that self-reported exposure estimates have more measurement error than "objective" estimates [55]. In general, this will lead to an underestimation of the true risk. However, if more than one risk factor in a multivariate analysis is measured with error, as is the case in this study, both underestimation and overestimation may occur [59]. It follows that our analysis on the contribution of various risk factors might be constrained by differences in measurement accuracy.

### Interpretation of epidemiological findings

It has been suggested that information on physiological mechanisms is necessary to interpret epidemiological findings, since epidemiological data will always be compatible with a wide variety of underlying causal mechanisms [60]. The integration of epidemiological and physiological data might enhance the identification of causal risk factors. However, a wide range of mechanisms has been proposed to explain hand, arm, shoulder and neck symptoms among office workers, making it difficult to identify the mechanism(s) at stake. Both peripheral tissue injury and reorganization of the central nervous system have been put forward as potential mechanisms [61]. A substantial part of pathophysiological research has focused on sustained low intensity muscle activity, leading to muscle disorders and consecutive symptoms. Although supportive evidence for this mechanism is available [62], it has been criticized as well [63]. Since empirical tests of different theories are not available, the specific physiologic mechanism(s) underlying hand, arm, shoulder and neck symptoms cannot be identified yet. A possibility for further investigation is to concurrently test the predictions of different theories in epidemiological studies. According to Barr and co-workers [61], a high repetition of movements is the main risk factor. According to Visser and co-workers [62], continuous low intensity muscle contraction (caused by occupational mechanical exposure and dependent on different situational and individual factors) is the risk factor of main interest. Knardahl [63] proposes mental demands (i.e. information processing demands) during computer use, as the most important risk factor. To concurrently test these theories, the amount of unique variation in the health outcome explained by the different constructs and the amount explained by shared variance could be investigated [64]. Therefore, subgroups of workers should be defined, in which an increased risk is expected based on the constructs used in the theories. Moreover, the extent of improvement in the prediction of the health outcome by adding a construct from an alternative theory should be investigated. This improvement in prediction could be operationalized as the improvement in explained variance or the presentation of extra relevant attributable fractions for the added construct. We will use this approach in the PROMO study.

Indirect evidence of causality might also be attained from the results of primary preventive interventions. However, only a small number of such studies has been published so far [65-67], making inferences based on this kind of evidence premature.

To increase our understanding of the etiology of hand, arm, shoulder and neck symptoms among office workers, the best way forward might be to combine multidisciplinary research efforts of observational and (lab- and field-based) experimental research. Hypotheses stemming from different research traditions should be tested (concurrently) under different situations, to make inferences about specificity and generalizability. It follows that the evidence from different levels of inference (e.g. genetics, physiology, biomechanics, individual behaviour and culture) should be integrated to optimally serve the goals of public and occupational health [68]. For a start, in the PROMO study both lab-based researchers and field-based researchers will collaborate. Moreover, a range of different hypotheses will be used to interpret the collected data.

## Competing interests

The author(s) declare that they have no competing interests.

## Authors' contributions

All authors contributed to the design of the study. SIJ is the principle researcher and coordinates the acquisition of data. BB, AB, WM and PB supervise the study. SIJ made the initial draft. All authors provided comments on the draft versions and approved the final manuscript.

## Funding

This article was prepared as part of a PhD project within the framework of the Body@Work TNO VUmc research centre. The VU University medical centre and TNO Quality of Life fund Body@Work TNO VUmc. No external funding was obtained for this article.

## Pre-publication history

The pre-publication history for this paper can be accessed here:



## Figures and Tables

**Table 1:** inclusion criteria

1. The job function of the workers is "office worker". Main tasks are computer use, participation in meetings, giving presentations, reading and phoning.

2. Workers have an individual e-mail address at work
